# Influences of Process Parameters of Near-Field Direct-Writing Melt Electrospinning on Performances of Polycaprolactone/Nano-Hydroxyapatite Scaffolds

**DOI:** 10.3390/polym14163404

**Published:** 2022-08-19

**Authors:** Zhijun Chen, Yanbo Liu, Juan Huang, Ming Hao, Xiaodong Hu, Xiaoming Qian, Jintu Fan, Hongjun Yang, Bo Yang

**Affiliations:** 1 State Key Laboratory of Separation Membranes and Membrane Processes, School of Textile Science and Engineering, Tiangong University, Tianjin 300387, China; 2 State Key Laboratory of New Textile Materials and Advanced Processing Technologies, School of Textile Science and Engineering, Wuhan Textile University, Wuhan 430200, China; 3 School of Materials Science and Engineering, Wuhan Textile University, Wuhan 430200, China

**Keywords:** near-field direct-writing melt electrospinning, composite scaffolds, tissue engineering

## Abstract

In this paper, near-field direct-writing melt electrospinning technology was employed to fabricate a polycaprolactone/nano-hydroxyapatite (PCL/nHA) scaffold for future applications in tissue engineering. The influences of different fabrication parameters on the structural characteristics, mechanical properties, and thermal stability of the scaffolds were discussed. It was found that the moving speed of the receiving plate had the most significant effect on the scaffold performance, followed by the receiving distance and spinning voltage. The results also showed that these process parameters affected the fiber diameter, corresponding coefficient of variation, porosity of the composite scaffolds, and mechanical properties of the samples, including the tensile strength and fiber peeling strength. Moreover, the process parameters could influence the thermal degradation performance and melting process. Although the mass loss of the composite scaffolds was not obvious after degradation, the mechanical performance degraded severely. It was concluded that the more appropriate process parameters for preparing PCL/nHA scaffolds were a spinning voltage of −4 kV, receiving distance of 4 mm, moving speed of receiving plate of 5 mm/s, and melt temperature of 130 °C. This study proved that near-field direct-writing melt electrospinning technology is a good method to obtain PCL/nHA composite scaffolds with an excellent mechanical properties and desired morphology for future tissue engineering applications.

## 1. Introduction

Near-field direct-writing melt electrospinning (NFDWME) technology has attracted great attention because of its many advantages, such as its environment-friendly process, high speed, and shape controllability [1,2,3,4,5]. By introducing the high-voltage electrostatic field into precise 3D printing equipment, NFDWME is able to prepare an orderly arranged micro-nano fiber structure as desired [6,7,8,9,10,11]. Basically, the melt spinning jet moves linearly if the distance between the spinning needle and the receiving plate is reduced properly; the molten polymer is thus deposited orderly as fibers on the receiving plate, which is precisely controlled and moves as pre-programmed.

Benefitting from a wide variety of polymer options and consistent throughput, NFDWME has gradually extended its application in tissue engineering scaffolds recently. As a branch of tissue engineering, bone tissue engineering aims to repair bone defects by growing seed cells on biological scaffolds to form cell-scaffold complexes and fill the bone defects [12,13,14,15,16,17]. There is great potential for the clinical treatment of bone tissue reconstruction caused by trauma, tumor, deformity, and degeneration, among other reasons [18,19,20,21].

Polycaprolactone (PCL) is a synthetic polyester biopolymer material with a strong crystallinity and good flexibility. As a biomaterial that has been approved by the US FDA for tissue engineering, it has a good biocompatibility, biodegradability, and plasticity. However, it is not suitable for osteoblast adhesion or bone tissue regeneration because it has no biological activity and has a poor mechanical strength, slow degradation rate, smooth surface, and strong hydrophobicity [22,23,24]. Thus, it is usually utilized in combination with one or more other biomaterials in order to enhance the osteogenic properties and biomechanical strength. Nano-hydroxyapatite (nHA), a ceramic inorganic biological material, is often used as one of the options for improving the biological activity and mechanical strength of PCL, owing to its chemical composition and crystal structure being similar to those of natural bone minerals. Thus, it has good inductive and conductive osteogenic properties and a sufficient mechanical strength, which can promote the adhesion of osteoblasts and bone tissue deposition. However, there are some disadvantages, such as a low fatigue strength, poor flexibility and plasticity, easy agglomeration, and a burst effect when releasing drugs if nHA is used alone. Therefore, it is necessary to mix nHA with other materials to obtain better biological properties [25,26].

In order to fabricate PCL/nHA scaffolds with a high porosity for cell penetration, nutrient transmission, and cell excrement discharge, as well as a sufficient bonding force between the fibers to prevent the dislocation and slippage of fibers during the cell culture process [27,28,29,30,31], near-field direct-writing melt electrospinning technology has been used to form gridding scaffold composed of parallel PCL/nHA micro-filaments. Here, the influences of parameters such as spinning voltage, receiving distance, moving speed of receiving plate, and melt temperature on the performances of composite scaffolds have been discussed in detail, in order to explore a more appropriate preparation process of PCL/nHA scaffolds.

## 2. Experimental

### 2.1. Experimental Materials

PCL (relative average molecular weight 80,000 kDa) was purchased from Shanghai Aladdin Biochemical Technology Co., Ltd. (Shanghai, China). The nHA (relative average molecular weight 500 kDa) used in this work was obtained from Shanghai Aladdin Biochemical Technology Co., Ltd. (Shanghai, China). The purity of the nHA was not less than 97% and the particle size was less than 100 nm. Proteinase K with a relative molecular weight of about 29.3 kDa was produced by Yisheng Biotechnology (Shanghai, China) Co., Ltd. Sterile Tris-HCl buffer solution with a concentration of 1 mol/L and pH value of 8.8 was prepared by Shanghai Yuanye Biotechnology Co., Ltd. (Shanghai, China). Ethanol (analytical reagent) was purchased from Sinopharm Chemical Reagent Co., Ltd. (Shanghai, China).

### 2.2. Preparation of PCL/nHA Scaffolds

**Figure 1:**
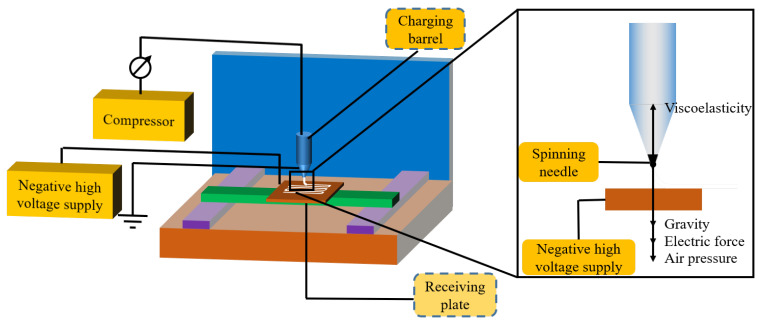
Schematic diagram showing the preparation of the PCL/nHA scaffolds.

The PCL/nHA scaffolds were prepared with near-field direct-writing melt electrospinning equipment (Qingzi Nano Co., Ltd. M08, Foshan China). The device mainly consisted of a compressor, a charging barrel with a spinning needle, and a receiving plate to collect the obtained fibers, as shown in Figure 1. The mixture of PCL and nHA was fed into the barrel to be heated and melted, forming a flowable polymer. The electric field exerted a force on the flowable polymer to form a Taylor cone. When the force exceeded the surface tension of the melt polymer, a liquid jet was extruded and accelerated towards the receiving plate. As the melt cooled in the air gap, a fiber formed, while simultaneously incurring Rayleigh axisymmetric, electric field-induced axisymmetric, and bending instabilities. By reducing the air gap distance coupled with precise translational movement between the polymer source and the receiving plate, individual electrospun fibers could be controlled to be directly “written” on the receiving plate, creating specific geometries that could be laid down layer by-layer. Thus, NFDWME allowed for fabricating desirable scaffolds with custom programmed geometries consisting of open interconnected pores and perfectly aligned fibers. In the present work, the fibers were first “written” in rows to prepare a horizontal layer, and then in columns to create a vertical layer, together forming a thin layer of a grid structure. The thickness of the final scaffold strongly relied on the number of grid layers.

Details of the PCL/nHA scaffolds fabricated with different processing parameters are presented in Table 1. Based on our previous trials, the mass ratio of PCL to nHA was selected as 90:10, which ensured the successful and continuous formation of PCL filaments. The inner diameter of the needle was 0.5 mm, the melt push force was 0.2 MPa, and the fiber spacing was set at 1.0 mm for each sample.

In addition, other fixed parameters included 1.0 mm for the fiber spacing, 0.5 mm for the needle inner diameter, and 0.2 MPa for the melt push force.

### 2.3. Testing

#### 2.3.1. Macroscopic Morphology

The sample size of the PCL/nHA scaffolds was 2 cm × 2 cm, and the macroscopic appearance of the sample was photographed with a camera.

#### 2.3.2. Fiber Diameter Based on SEM

After gold spraying, the surface morphology and cross section of the sample was observed using a scanning electron microscope (Japan Electronics Co., Ltd. JSM-6510LV, Tokyo, Japan). Fifty fibers were randomly selected using Image J software (ImageJ 1.8.0.172, National Institutes of Health, Bethesda, Maryland, USA) to measure and calculate the fiber diameter.

#### 2.3.3. Porosity

The length, width, and height of the PCL/nHA sample were measured, and the volume was recorded as *V*_1_. A certain amount of ethanol was added into a graduated cylinder, and the volume was recorded as *V*_2_. The sample was immersed in ethanol for 10 min to ensure no bubbles on the sample surface, and the volume was recorded as *V*_3_. The porosity (ε) could be calculated using Equation (1):

*ε* = [1 − (*V*_3_ − *V*_2_)/*V*_1_] × 100% (1)

#### 2.3.4. Mechanical Performance

The mechanical performance of PCL/nHA scaffolds with a length of 20 mm and width of 5 mm was investigated with an electronic universal testing machine (MTS Systems CO., LTD E44.104, Shanghai, China). The length gauge was 10 mm and the tensile speed was 10 mm/min. Five measurements were taken in each group and the results were averaged.

#### 2.3.5. Fiber Peeling Strength

As the extruded fibers were laid down layer by layer, the subsequently formed fibers bonded with the previous fiber layers, and the adhesion force between the fibers played an important role in determining the strength of the scaffold. The peeling strength between a single fiber was employed to characterize the adhesion behavior between the fiber. Tweezers were used to pick out a single fiber and clamp it in the upper clamp of an electronic universal testing machine (MTS Systems CO., LTD E44.104, Shanghai, China), while the lower end of the sample was clamped in the lower clamp. The drawing speed was set at 10 mm/min. The fiber was peeled from the surface of the sample as the upper clamp moved up, and the maximum tensile force when the fiber completely detached from the sample was recorded.

#### 2.3.6. Thermal Stability

(1) Thermogravimetric (TG) analysis

The sample with a weight of 2–3 mg was tested with a TG analyzer (TA instrument TGA55, New Castle, Delaware, USA) in order to investigate the thermal performance. The temperature range was 30–600 °C, the heating rate was 10 °C/min and the nitrogen flow rate was 40 mL/min.

(2) Differential scanning calorimeter (DSC) analysis

The sample with weight of 4 mg was tested with a DSC (TA instrument DSC25, New Castle, DE, USA). The temperature raised from 30 °C to 150 °C at a rate of 10 °C/min for the first time, and remained at 150 °C for 5 min under a nitrogen atmosphere; the sample was subsequently cooled to −20 °C at a rate of −10 °C/min, held at −20 °C for 5 min, followed by heating up to 150 °C at a rate of 10 °C/min again, and then cooled to room temperature.

#### 2.3.7. Degradability

Proteinase K was dissolved in a Tris-HCl buffer solution to prepare a degradation solution with a proteinase K concentration of 0.4 mg/mL. The PCL/nHA scaffolds were suspended in a test tube containing the degradation solution and were placed in a shaking water bath at a constant temperature at 37 °C. Samples were taken out and weighed every two days, and the mass loss rate of the sample was calculated. The relationship between the degradation time and the mass loss rate was obtained accordingly.

## 3. Results and Discussion

### 3.1. Influence of Process Parameters on the Morphology of PCL/nHA Scaffolds

The formation of fibers and the layout of fiber layers strongly depends on the process parameters of near-field direct-writing melt electrospinning technology, such as the spinning voltage, receiving distance, moving speed of the receiving plate, and melt temperature. The effects of these parameters on the morphology of the PCL/nHA scaffolds were studied and analyzed based on a single-factor experiment.

#### 3.1.1. Influence of Spinning Voltage on the Morphology of PCL/nHA Scaffolds

**Figure 2:**
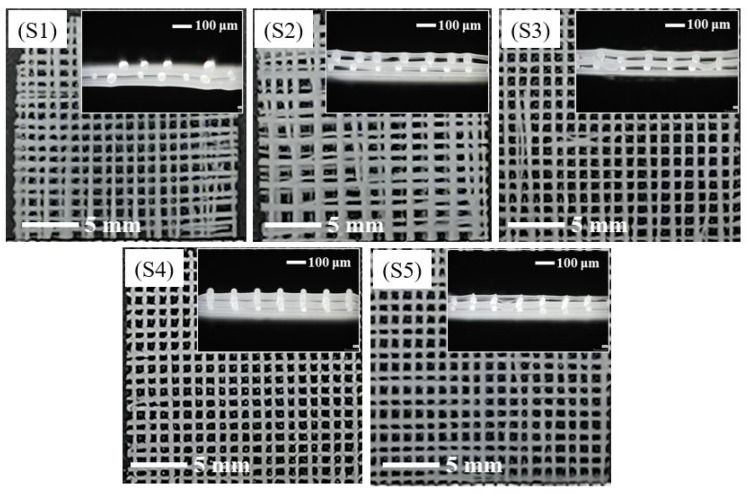
The surface morphologies and cross-sectional views of PCL/nHA scaffolds with various voltages: voltages of samples **S1**–**S5** are 0, −2, −3, −4, and −5 kV, respectively.

The surface morphology and cross-sectional views of the PCL/nHA scaffolds are shown in Figure 2. The voltages of these samples were 0, −2, −3, −4, and −5 kV, respectively, as listed in Table 1, with a fixed receiving distance 4 mm, moving speed of the receiving plate of 5 mm/s, and melt temperature of 130 °C. It was found that the scaffolds had a grid morphology with a clear and interconnected pore structure. The upper and lower fibers in the grid crossed vertically, and the transverse and longitudinal fibers were arranged regularly. In the preparation process, the extruded polymer suffered from the electric field force, gravity, and pulling force of the platform movement. The electric field force caused by the electrostatic voltage between the spinning jet and the receiving plate was an important drawing force during spinning. The higher the voltage, the greater the charge density on the melt surface, providing a stronger electric field force for fiber stretching to obtain thinner and more even fiber, as well as a more powerful bonding force between the fibers. When the voltage was zero, there was no electric field force at all. The gravity and pulling force were insufficient to draw the fibers, resulting in a thicker fiber, irregular fiber deposition, and small bonding force between fibers. When the voltage increased to −3 kV, the electric field force increased as well. However, it was still insufficient to fully stretch the fibers, causing less continuous fibers and unparalleled arrangement of the fiber layers. When the voltage was −4 kV, the transverse and longitudinal fibers were arranged regularly, and the scaffold pores were neat square cells with the same size, as shown in Figure 2(S4). When the voltage was −5 kV, the scaffold structure showed a stagger arrangement of small pores and relatively larger pores, as seen in Figure 2(S5), and the distance between the two adjacent fibers appeared to alternate in a manner of “near/far”. This could be explained by the influence of the attraction of the electrostatic force of the fibers. When one fiber fell, it was pushed away by the repulsive force due to the electron distributed on the fiber surface, and the falling fiber deviated from the trajectory where it should have been deposited, resulting in closer spacing of the two fibers. When another fiber fell, the neighboring fiber was slightly farther away, and the electrostatic force was weak, causing a farther fiber depositing. Repeatedly, the neighboring fibers were alternatively arranged in this way. However, the power supply self-protected and stopped working when the voltage further increased to −6 kV.

#### 3.1.2. Influence of Receiving Distance on the Morphology of PCL/nHA Scaffolds

**Figure 3:**
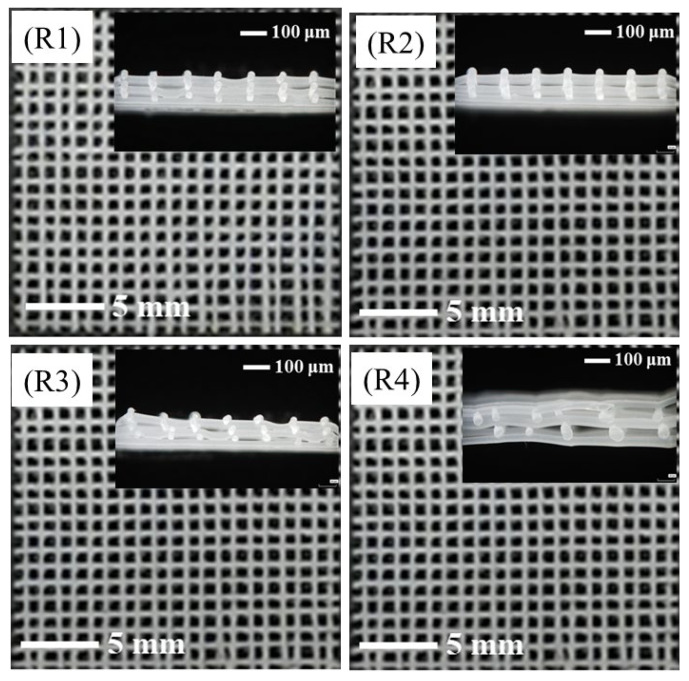
The surface morphologies and cross-sectional views of the PCL/nHA scaffolds prepared with various receiving distances: the receiving distances of samples **R1**–**R4** are 3, 4, 5, and 6 mm, respectively.

The receiving distance, also known as the distance between the spinning needle and the receiving plate, determines the travelling time of the polymer fibers in the electrostatic field. Generally, the larger the receiving distance, the longer the travelling time, and thus the stronger the fiber bonding. The morphologies of the PCL/nHA scaffolds prepared with different receiving distances are illustrated in Figure 3. The receiving distances were selected as 3, 4, 5, and 6 mm, respectively, with a constant spinning voltage of −4 kV, moving speed of the receiving plate of 5 mm/s, and melt temperature of 130 °C. Because of the self-protection of the power supply and short circuit, the equipment was not able to work when the receiving distance was lower than 3 mm. As the receiving distance increased to 3 and 4 mm, the molten polymer stretched in the electric field and cooled down to form continuous fibers, creating regular fiber deposition with uniform fiber diameters. At a receiving distance of 5 mm, the fiber regularity became worse, and the fiber diameter was not uniform, resulting in inconsistent thickness of the samples in Figure 3(R3). The polymer fiber could not be deposited at the designated position in a limited time, causing deterioration of the regularity of the fiber deposition for a receiving distance of 6 mm, as shown in Figure 3(R4). The above results demonstrate that the near-field direct-writing melt electrospinning required a certain receiving distance to ensure that the fiber could be fully stretched under the action of the electric field force during the falling process, which plays a role in fiber refinement. However, the receiving distance could not be increased indefinitely, because the increase in the receiving distance weakened the electric field force on the fibers, causing instability of the fiber formation, which was not conducive to accurate fiber deposition.

#### 3.1.3. Influence of Moving Speed of the Receiving Plate on the Morphology of PCL/nHA Scaffolds

**Figure 4:**
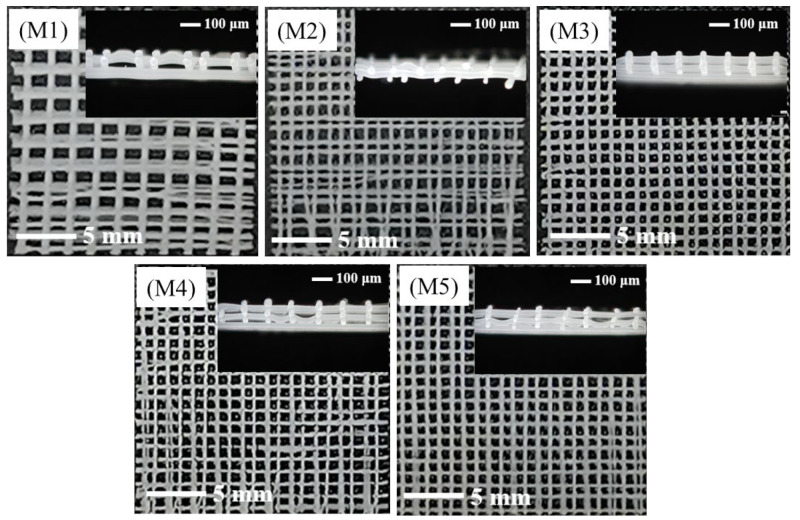
The surface morphologies and cross-sectional views of the PCL/nHA scaffolds prepared with various moving speeds of the receiving plate: moving speeds of receiving plate of samples **M1**–**M5** are 3, 4, 5, 6, and 7 mm/s, respectively.

As shown in Figure 4, the moving speeds of the receiving plate used to prepare the samples of M1–M5 were 3, 4, 5, 6, and 7 mm/s, respectively. The moving speed of the receiving plate determined the pulling force on the fiber, which had a great influence on the fiber fineness and the uniformity of the fiber diameter. When the moving speed was 3 or 4 mm/s, the fiber movement was slow and more polymers were deposited per unit time; thus, the fiber was not effectively drawn, forming thick fibers, and these fibers crowded together or even merged. Consequently, the regularity of the scaffold was poor. If the speed was too high, the fiber was subjected to a large pulling force that had to be attenuated in a short time. Hence, the uniformity of the fiber thickness was not high, as seen in Figure 4(M4).

#### 3.1.4. Influence of Melt Temperature on the Morphology of PCL/nHA Scaffolds

**Figure 5:**
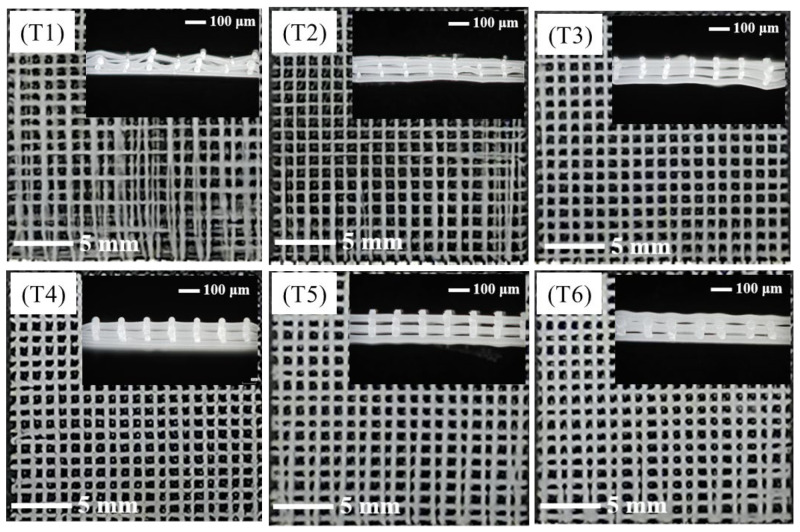
The surface morphologies and cross-sectional views of PCL/nHA scaffolds with various melt temperatures: melt temperatures of samples **T1**–**T6** are 100, 110, 120, 130, 140, and 150 °C, respectively.

PCL/nHA scaffolds were prepared under melt temperatures of 100, 110, 120, 130, 140, and 150 °C in order to investigate the effect of melt temperature on the scaffold morphology in Figure 5. When the melt temperature was 100 °C, the melt had a poor fluidity due to s very high viscosity, contributing to the formation of thin and uneven fibers stretched by the continuous pulling force of the receiving platform. After increasing the melt temperature to 110 °C or 120 °C, the regularity of the scaffold was obviously improved, but the fiber fineness was still uneven. At a melt temperature of 130 °C, the fiber thickness was uniform and the scaffold had a high degree of regularity. At 140 °C, the melt fluidity was very high, which allowed for better extension of the polymer in the electrostatic field. The high temperature also extended the solidification time of the fibers, so that the fibers were thicker at intersections and thinner between intersections, which caused worse fiber uniformity than the sample at a melt temperature of 130 °C. When the melt temperature reached 150 °C, it required a longer time to fully cool the fibers as the upper and lower fibers were stacked together. Meanwhile, the shape of the fiber changed from a circle to dumbbell with the increase in melt temperature due to the excellent melt liquidity under a high temperature. The flowing of the melt in the extended solidification time of the polymer with a high temperature caused the formation of a dumbbell shape.

### 3.2. Fiber Diameter and Porosity of PCL/nHA Scaffolds

The average value and coefficient of variation (CV) of fiber diameter and porosity of the PCL/nHA scaffolds are exhibited in Table 2. The fiber diameter decreased with the increase in spinning voltage and melt temperature. When the spinning voltages were 0 and −2 kV, the CV of the fiber diameter were more than 15%, indicating that the fiber was uneven, which was consistent with the analysis result in Section 3.1.1. The receiving distance had an insignificant effect on the fiber diameter. It mainly affected whether the fibers were orderly deposited, rather than the fiber fineness. At a receiving distance of 5 mm, the fiber regularity was observed to become worse. The moving speed of the receiving plate determined the pulling force on the fiber. When the moving speed was 3 mm/s, the fiber was insufficiently stretched and the average fiber diameter was 360 μm. The larger the moving speed of the receiving plate, the smaller the fiber diameter, but the larger the CV of the fiber diameter and the greater the unevenness of fiber thickness. Based on a comparative analysis of the influences of various process parameters on the porosity of PCL/nHA scaffolds, it was found that the porosity was mainly distributed between 70% and 80%, and there was no obvious regularity, which suggested that the manufacturing process had little influence on the porosity of the PCL/nHA scaffolds.

### 3.3. Mechanical Properties of PCL/nHA Scaffolds

**Figure 6:**
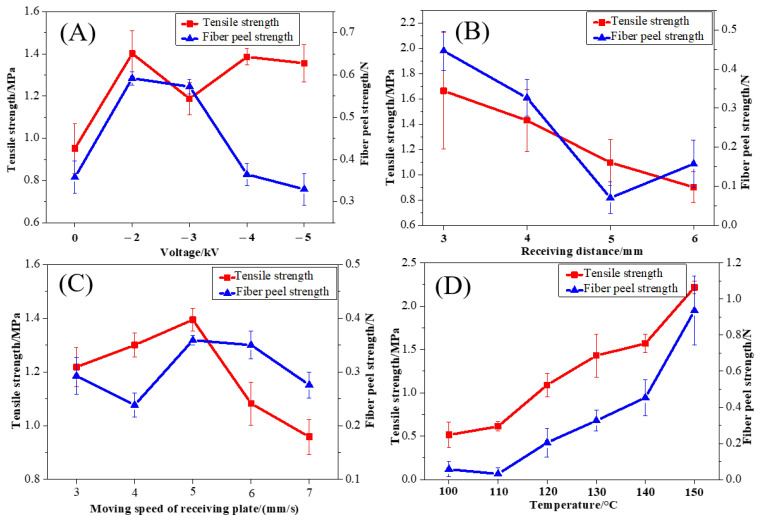
The influences of the process parameters on the mechanical properties of PCL/nHA scaffolds: (**A**) voltage, (**B**) receiving distance, (**C**) moving speed of the receiving plate, and (**D**) temperature.

The scaffold regularity and the uniformity of the fiber diameter had a great impact on the tensile strength of the sample. The fiber peeling strength was used to characterize the bonding force of the fibers between layers. A large fiber peeling strength implied high regularity of the sample, strong bonding at the fiber intersections, and stable mechanical properties of the scaffolds. The tensile strength and fiber peeling strength of the PCL/nHA scaffolds are shown in Figure 6. As shown in Figure 6A, as the voltage increased, the tensile strength of the PCL/nHA scaffolds tended to increase initially and decrease afterwards. Owing to the greater drawing of the fibers under a larger voltage, the fibers were not fully orientated and crystallized, resulting in poor fiber bonding and thus a reduced fiber peeling strength. In Figure 6B, as the receiving distance increased, both the tensile strength and fiber peeling strength of the scaffolds decreased. The greater the receiving distance, the longer the cooling time of the fiber and the weaker the bonding force between the fibers. As the moving speed of the receiving plate increased, the tensile strength and the fiber peeling strength of the PCL/nHA scaffolds increased first and then decreased, as seen in Figure 6C. It was obvious that the PCL/nHA scaffolds had a relatively high tensile strength of 1.39 MPa and a good fiber peeling strength of 0.36 N at a suitable speed of 5 mm/s. At a slow-moving speed of the receiving plate, the prepared fibers were thin and uneven, showing a lower fiber peeling strength. However, at a fast-moving speed of the receiving plate, the fiber bonding became weak, which also contributed to poor mechanical properties. As shown as Figure 6D, with the increase in melt temperature, the tensile strength and fiber peel strength increased significantly. When the melt temperature was 150 °C, the tensile strength and fiber peeling strength reached a maximum of 2.22 MPa and 0.94 N, respectively. This indicated that the melt temperature had a significant effect on the tensile strength and fiber peeling strength of the PCL/nHA scaffolds, owing to the strong bonding of the fibers at a high temperature. Taking the morphology, fiber diameter, and porosity of the PCL/nHA scaffolds into consideration, the optimal process parameters to obtain an excellent comprehensive performance were −4 kV voltage, 4 mm receiving distance, 5 mm/s moving speed of the receiving plate, and 130 °C melt temperature.

### 3.4. Thermal Propreties of PCL/nHA Scaffolds

Three PCL/nHA scaffolds prepared under melt temperatures of 110, 130, and 150 °C were selected for both the TG analysis and DSC analysis.

**Figure 7:**
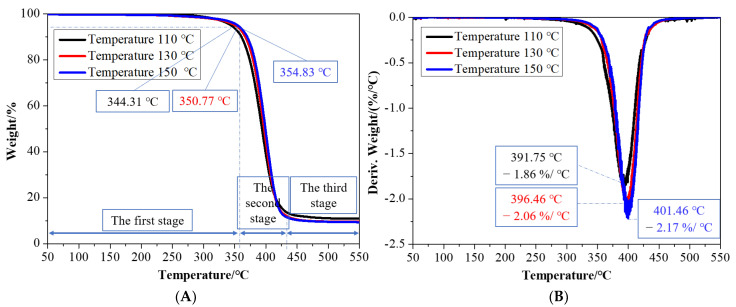
TG (**A**) and DTG (**B**) analysis of the PCL/nHA scaffolds.

The TG curves of the PCL/nHA scaffolds with different melt temperature are shown in Figure 7. The thermal degradation of the PCL/nHA scaffolds under a nitrogen atmosphere was divided into three stages. The first stage, at 30–355 °C, was the mass loss of small molecules, such as water, in the sample; 5% mass loss was observed at 344.31 °C, 350.77 °C, and 354.83 °C, separately. The second stage, at 355–430 °C, was the rapid degradation stage of PCL/nHA scaffolds. In the third stage, at 430–550 °C, the samples were basically degraded, and finally only residue remained. The residue contents of the three samples with the same composition were about 10%.

**Figure 8:**
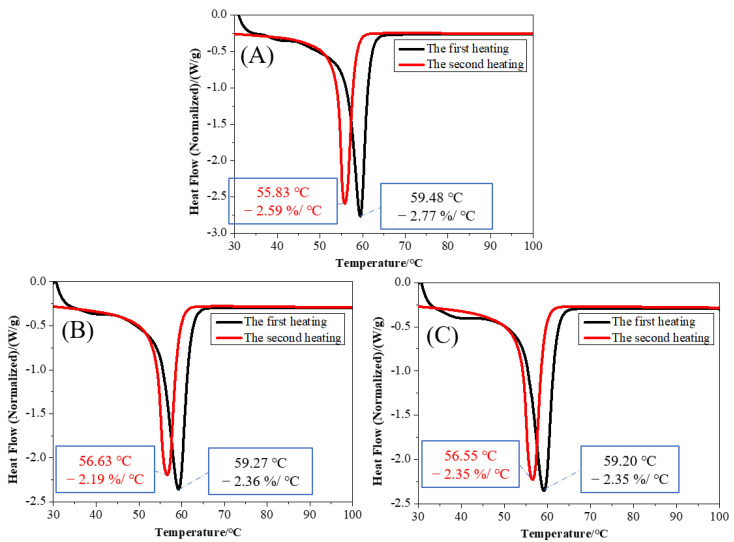
DSC comparisons of the first heating and the second heating curves of the PCL/nHA scaffolds with different melt temperatures: (**A**) 110 °C, (**B**) 130 °C, and (**C**) 150 °C.

The comparisons between DSC curves of the first heating and the second heating of the three samples are shown exhibited in Figure 8. It can be seen that the DSC curves of the three samples were basically the same at the first heating stage. The peak value of the second heating curve was smaller than the peak value of the first heating curve. After the first melting and cooling, the second melting peak of the sample decreased, and the corresponding endothermic enthalpy also decreased.

### 3.5. Degradability of PCL/nHA Scaffolds

**Figure 9:**
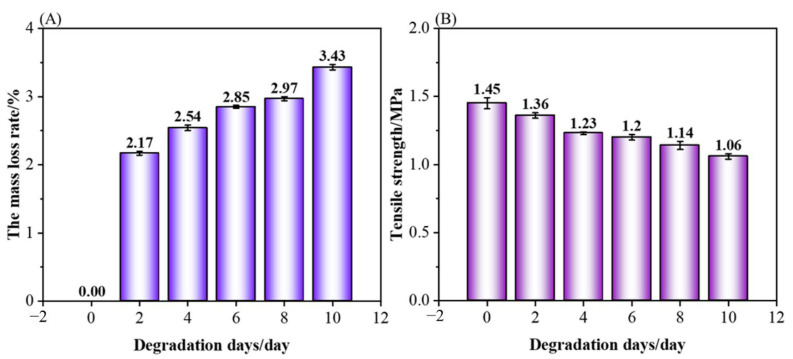
Mass loss rate and tensile strength of the PCL/nHA scaffolds before and after degradation: (**A**) mass loss rate, (**B**) tensile strength.

The changes in the weight and tensile strength of the PCL/nHA scaffolds before and after treatment in proteinase K solution for 2, 4, 6, 8, and 10 days are shown in Figure 9. From Figure 9A, the mass loss rate of the PCL/nHA scaffolds after two days of degradation was 2.17%, and that of the PCL/nHA scaffolds increased as the days of degradation increased, indicating that the PCL/nHA scaffolds after treatment in a proteinase K solution could be efficient for degradation. In Figure 9B, the tensile strength of the scaffolds without degradation was 1.45 MPa, while the tensile strength of the PCL/nHA scaffolds after two days and ten days of degradation were 1.36 MPa and 1.06 MPa, respectively. The tensile strength of the scaffolds after ten days of degradation decreased by 73.10% of that of the scaffolds without degradation. Generally, at the beginning of the degradation of PCL/nHA scaffolds in the proteinase K solution, the binding of nHA and small debris from the fiber surface of the scaffolds would be loose and degraded, and the structure of PCL nanofibers, as the main bearing structure, was rarely broken. As the days of degradation increased, main bearing structure of the PCL/nHA scaffolds began to decompose and break. Therefore, the PCL/nHA scaffolds after treatment in the proteinase K solution had a good biodegradability.

## 4. Conclusions

The PCL/nHA scaffolds were prepared using near-field direct-writing melt electrospinning technology with different process parameters, such as spinning voltage, receiving distance, moving speed of the receiving plate, and melt temperature. The surface morphology, cross-sectional view, fiber diameter, porosity, mechanical properties, and thermal properties of the PCL/nHA scaffolds were characterized and analyzed. The process parameters had a certain effect on the structural characteristic of the scaffolds, including the fiber diameter, uniformity of the fiber diameter, and porosity of the scaffolds. Meanwhile, these parameters played a significant role in the tensile strength and fiber peeling strength, and had a certain impact on the thermal degradation performance. Based on the comprehensive comparison and analysis, the optimal process parameters for preparing the PCL/nHA scaffolds were as follows −4 kV voltage, 4 mm receiving distance, 5 mm/s moving speed of the receiving plate, and 130 °C melt temperature. This work proved that it was possible to prepare scaffold for tissue engineering with a designed shape at a high speed using near-field direct-writing melt electrospinning technology. The relationship between the process parameters and scaffold performance was investigated, which is important for the design of scaffolds with an appropriate physical performance and degradation rate. In the future, the biocompatibility of PCL/nHA scaffolds should be investigated and discussed, which will offer certain guidance in practice for tissue engineering and clinical applications.

## Figures and Tables

**Table 1:** Details of the PCL/nHA scaffolds fabricated with different processing parameters.

Sample Code	Process Parameters			
	Spinning Voltage (kV)	Receiving Distance (mm)	Moving Speed of Receiving Plate (mm/s)	Melt Temperature (°C)
S1	0	4	5	130
S2	−2	4	5	130
S3	−3	4	5	130
S4	−4	4	5	130
S5	−5	4	5	130
R1	−4	3	5	130
R2	−4	4	5	130
R3	−4	5	5	130
R4	−4	6	5	130
M1	−4	4	3	130
M2	−4	4	4	130
M3	−4	4	5	130
M4	−4	4	6	130
M5	−4	4	7	130
T1	−4	4	5	100
T2	−4	4	5	110
T3	−4	4	5	120
T4	−4	4	5	130
T5	−4	4	5	140
T6	−4	4	5	150

**Table 2:** Fiber diameter and porosity of the PCL/nHA scaffolds.

Parametric Variable	Parameter	Average of Fiber Diameter/μm	CV Value of Fiber Diameter/%	Porosity/%
Spinning voltage	0	200	17.49	76.7
	−2 kV	285	15.34	75.1
	−3 kV	215	6.56	79.8
	−4 kV	224	5.39	78.3
	−5 kV	225	7.67	80.2
Receiving distance	3 mm	212	7.07	76.5
	4 mm	224	5.39	78.3
	5 mm	194	10.17	81.0
	6 mm	197	12.89	80.5
Moving speed of receiving plate	3 mm/s	360	23.50	73.5
	4 mm/s	275	9.52	78.0
	5 mm/s	224	5.39	78.3
	6 mm/s	197	11.14	82.1
	7 mm/s	193	12.36	80.2
Melt temperature	100 °C	157	11.79	84.8
	110 °C	182	13.62	82.8
	120 °C	207	11.31	80.3
	130 °C	224	5.39	78.3
	140 °C	273	5.98	73.4
	150 °C	299	6.48	66.5

## References

[1] Chen Z., Hao M., Qian X., Chen W., Zeng M., Huang J., Li R., Fan J., Liu Y. (2021). Characterization on Modification and Biocompatibility of PCL Scaffold Prepared with Near-field Direct-writing Melt Electrospinning. Chem. Res. Chin. Univ..

[2] Meng J., Boschetto F., Yagi S., Marin E., Adachi T., Chen X., Pezzotti G., Sakurai S., Yamane H., Xu H. (2021). Design and manufacturing of 3D high-precision micro-fibrous poly (l-lactic acid) scaffold using melt electrowriting technique for bone tissue engineering. Mater. Des..

[3] Jin Y., Gao Q., Xie C., Li G., Du J., Fu J., He Y. (2020). Fabrication of heterogeneous scaffolds using melt electrospinning writing: Design and optimization. Mater. Des..

[4] Nazemi M.M., Khodabandeh A., Hadjizadeh A. (2022). Near-Field Electrospinning: Crucial Parameters, Challenges, and Applications. ACS Appl. Bio Mater..

[5] Daghrery A., de Souza Araújo I.J., Castilho M., Malda J., Bottino M.C. (2022). Unveiling the potential of melt electrowriting in regenerative dental medicine. Acta Biomater..

[6] Brown T.D., Dalton P.D., Hutmacher D.W. (2011). Direct Writing By Way of Melt Electrospinning. Adv. Mater..

[7] Ilhan E., Cesur S., Guler E., Topal F., Albayrak D., Guncu M.M., Cam M.E., Taskin T., Sasmazel H.T., Aksu B. (2020). Development of Satureja cuneifolia-loaded sodium alginate/polyethylene glycol scaffolds produced by 3D-printing technology as a diabetic wound dressing material. Int. J. Biol. Macromol..

[8] Mahendiran B., Muthusamy S., Sampath S., Jaisankar S.N., Popat K.C., Selvakumar R., Krishnakumar G.S. (2021). Recent trends in natural polysaccharide based bioinks for multiscale 3D printing in tissue regeneration: A review. Int. J. Biol. Macromol..

[9] Nguyen N.T., Kim J.H., Jeong Y.H. (2019). Identification of sagging in melt-electrospinning of microfiber scaffolds. Mater. Sci. Eng. C.

[10] Chen P., Cui L., Chen G., You T., Li W., Zuo J., Wang C., Zhang W., Jiang C. (2019). The application of BMP-12-overexpressing mesenchymal stem cells loaded 3D-printed PLGA scaffolds in rabbit rotator cuff repair. Int. J. Biol. Macromol..

[11] Wang X., Zheng G., Xu L., Cheng W., Xu B., Huang Y., Sun D. (2012). Fabrication of nanochannels via near-field electrospinning. Appl. Phys. A.

[12] Rezvani Z., Venugopal J.R., Urbanska A.M., Mills D.K., Ramakrishna S., Mozafari M. (2016). A bird’s eye view on the use of electrospun nanofibrous scaffolds for bone tissue engineering: Current state-of-the-art, emerging directions and future trends. Nanomed. Nanotechnol. Biol. Med..

[13] Babaie E., Bhaduri S.B. (2018). Fabrication Aspects of Porous Biomaterials in Orthopedic Applications: A Review. ACS Biomater. Sci. Eng..

[14] Adithya S.P., Sidharthan D.S., Abhinandan R., Balagangadharan K., Selvamurugan N. (2020). Nanosheets-incorporated bio-composites containing natural and synthetic polymers/ceramics for bone tissue engineering. Int. J. Biol. Macromol..

[15] Collins M.N., Ren G., Young K., Pina S., Reis R.L., Oliveira J.M. (2021). Scaffold Fabrication Technologies and Structure/Function Properties in Bone Tissue Engineering. Adv. Funct. Mater..

[16] Vacanti J.P., Langer R. (1999). Tissue engineering: The design and fabrication of living replacement devices for surgical reconstruction and transplantation. Lancet.

[17] Langer R., Vacanti J.P. (1993). Tissue Engineering. Science.

[18] Yadav L.R., Chandran S.V., Lavanya K., Selvamurugan N. (2021). Chitosan-based 3D-printed scaffolds for bone tissue engineering. Int. J. Biol. Macromol..

[19] Farrugia B.L., Brown T.D., Upton Z., Hutmacher D.W., Dalton P.D., Dargaville T.R. (2013). Dermal fibroblast infiltration of poly(ε-caprolactone) scaffolds fabricated by melt electrospinning in a direct writing mode. Biofabrication.

[20] Liu Y., Wang R., Chen S., Xu Z., Wang Q., Yuan P., Zhou Y., Zhang Y., Chen J. (2020). Heparan sulfate loaded polycaprolactone-hydroxyapatite scaffolds with 3D printing for bone defect repair. Int. J. Biol. Macromol..

[21] Li K., Zhang F., Wang D., Qiu Q., Liu M., Yu A., Cui Y. (2021). Silkworm-inspired electrohydrodynamic jet 3D printing of composite scaffold with ordered cell scale fibers for bone tissue engineering. Int. J. Biol. Macromol..

[22] Alonzo M., Alvarez Primo F., Anil Kumar S., Mudloff J.A., Dominguez E., Fregoso G., Ortiz N., Weiss W.M., Joddar B. (2021). Bone tissue engineering techniques, advances, and scaffolds for treatment of bone defects. Curr. Opin. Biomed. Eng..

[23] Zhao X., Lui Y.S., Choo C.K.C., Sow W.T., Huang C.L., Ng K.W., Tan L.P., Loo J.S.C. (2015). Calcium phosphate coated Keratin–PCL scaffolds for potential bone tissue regeneration. Mater. Sci. Eng. C.

[24] Großhaus C., Bakirci E., Berthel M., Hrynevich A., Kade J.C., Hochleitner G., Groll J., Dalton P.D. (2020). Melt Electrospinning of Nanofibers from Medical-Grade Poly(ε-Caprolactone) with a Modified Nozzle. Small.

[25] Kumar P., Saini M., Dehiya B.S., Umar A., Sindhu A., Mohammed H., Al-Hadeethi Y., Guo Z. (2020). Fabrication and in-vitro biocompatibility of freeze-dried CTS-nHA and CTS-nBG scaffolds for bone regeneration applications. Int. J. Biol. Macromol..

[26] Wang Z., Wang Y., Yan J., Zhang K., Lin F., Xiang L., Deng L., Guan Z., Cui W., Zhang H. (2021). Pharmaceutical electrospinning and 3D printing scaffold design for bone regeneration. Adv. Drug Deliv. Rev..

[27] Jiang T., Carbone E.J., Lo K.W.H., Laurencin C.T. (2015). Electrospinning of polymer nanofibers for tissue regeneration. Prog. Polym. Sci..

[28] Wu J., Hong Y. (2016). Enhancing cell infiltration of electrospun fibrous scaffolds in tissue regeneration. Bioact. Mater..

[29] Yang G., Li X., He Y., Ma J., Ni G., Zhou S. (2018). From nano to micro to macro: Electrospun hierarchically structured polymeric fibers for biomedical applications. Prog. Polym. Sci..

[30] Xiao L., Wu M., Yan F., Xie Y., Liu Z., Huang H., Yang Z., Yao S., Cai L. (2021). A radial 3D polycaprolactone nanofiber scaffold modified by biomineralization and silk fibroin coating promote bone regeneration in vivo. Int. J. Biol. Macromol..

[31] Xie C., Gao Q., Wang P., Shao L., Yuan H., Fu J., Chen W., He Y. (2019). Structure-induced cell growth by 3D printing of heterogeneous scaffolds with ultrafine fibers. Mater. Des..

